# Supplementary material for: Patterns of Intron Gain and Loss in Fungi
Source: PLoS Biol. 2004 Nov 30;2(12):e422. doi: 10.1371/journal.pbio.0020422 (PMC532390; doi:10.1371/journal.pbio.0020422)
Supplement: Table S1 — Also available at http://genes.mit.edu/NielsenEtAl/. (4.3 MB ZIP). [file pbio.0020422.st001.zip › NielsenEtAl/html/1437.html]

AN1133.1.NCU02463.1.MG03089.1.FG09422.1


```
 CLUSTAL W (1.82) Multiple Sequence Alignments - Introns Inserted


Sequence 1: MG03089.1	282 aa
Sequence 2: FG09422.1	276 aa
Sequence 3: NCU02463.1	287 aa
Sequence 4: AN1133.1	270 aa
Alignment Length: 288 aa
Number Identitical Residues: 145 aa
Alignment Score (without introns) 7043


MG03089.1 	MAANAKYTAAPTQDPDDHLDYTSAPPSYQATGSSAAQP---SDAERLASGVPRSSEDD--
NCU02463.1	-MSGPKYTPAPQEDPDAHLYYPQPPPSYQATAESSAANAVLGDQARLFSGAPRSSQDDDH
FG09422.1 	MASKTKYQTAPQSDPDD--DYTHAPPAYAEG--SAAR-----DETQGLFGAPRDSEDN--
AN1133.1  	MATNARYEPAPQRDSFEDQHYTQAPPSYQAT---AQEP------------TPRSEDDN--
          	  : .:* .**  *.     *. .**:*      :               .**..:*:  

MG03089.1 	-LPDDFK~FGGNVAEATVEIRHQFIRKVYSIL~TVQLLITGGVSALGFMSTSYRDWVRAH
NCU02463.1	DIPDDFK0FGGSVAEATVEIRHQFIRKVYTIL~TVQLIATGAVSALSFLSDGYRNWIQSH
FG09422.1 	-LPDDFK0FGGSVAEATVDIRNQFVRKVYTIL~TVQLLATAGVSSLTFFSTGYKDWIQSH
AN1133.1  	-VPDDFK0FGGTVAEGTLPIRMQFIRKVYAIL2TVQLLLTTVMSSISFFSPSYCEWIRSN
          	 :***** ***.***.*: ** **:****:** ****: *  :*:: *:* .* :*::::

MG03089.1 	PGVLWLS0LFGAMGMMLLTYWKRKSYPTNLLFLGGFTLLEAYTISVVVTFYDSSIVLNAV
NCU02463.1	PAMIWVS0FAGALVFMLLTFWKRQSYPTNLLFLSGFTLLEAYTISVCVSFFDSTTVLLAV
FG09422.1 	PGVVWAS0LFGSMIFMGLTYWKRKSYPTNLLFLSLFTLAEAYTISVIVSFYKTSIVLNAV
AN1133.1  	VWLMMVS~VFGAFGFLLVTYWKRKSYPANLLFLSAFTILEAYSISVVTSYYQPRIVVQAL
          	  ::  * . *:: :: :*:***:***:*****. **: ***:*** .:::..  *: *:

MG03089.1 	LLTAGMFVFLTAFAFQTKYDFTSWMPYLFGALWGLVLFGFVAMFLPYSSTAELIYGALAA
NCU02463.1	VITAGIFVFLTAFACQTKYDFTSWMPYLGGALWGLIITGFIYAFLPHTSTSELVYGGVAA
FG09422.1 	VLTAGIFVFLTLFACQTKYDFTSWMPYLFGALWGLVIFGFMAMFFPYSSTGELIYGGLAA
AN1133.1  	ILTLGLFVGLTLFACQTKYDFTNWMPYLFGALWFLILFGFVAAFVPHGSTMELIYGSLAA
          	::* *:** ** ** *******.***** **** *:: **:  *.*: ** **:**.:**

MG03089.1 	LVFSGYILVDTQLVMRTHHVEEEIAAAISLYLDIINLFLAILRILNSQQQN
NCU02463.1	LVFSGYILVDTQLVMRKYHVEEEIAAAISLYLDILNLFLAILRILNSQSDN
FG09422.1 	LIFSGYILVDTQLVLRHHHVEEEIAAAISLYLDIINLFLAILRILNSQSNN
AN1133.1  	LIFSGYILVDTQLIMRHYHVEEEIAASISLYLDILNLFLAILRILNNQNNN
          	*:***********::* :********:*******:***********.*.:*
```
